# Current prevalence of perioperative early venous thromboembolism and risk factors in Chinese adult patients with inguinal hernia (CHAT-1)

**DOI:** 10.1038/s41598-020-69453-6

**Published:** 2020-07-29

**Authors:** Minggang Wang, Guangyong Zhang, Jie Chen, Jianwen Li, Yan Che, Jianxiong Tang, Hangyu Li, Junsheng Li, Yingmin Ma

**Affiliations:** 1 Department of Hernia and Abdominal Wall Surgery, Beijing Chaoyang Hospital, Capital Medical University, Beijing, 100043 China; 2 Department of General Surgery, The First Affiliated Hospital of Shandong First Medical University, Jinan, 250012 China; 3 Department of General Surgery, Ruijin Hospital, Shanghai Jiao Tong University School of Medicine, Shanghai, 200020 China; 4 NHC Key Laboratory of Reproduction Regulation, Shanghai Institute of Planned Parenthood Research, Fudan University, Shanghai, 200020 China; 5 Department of General Surgery, Huadong Hospital Affiliated to Fudan University, Shanghai, 200040 China; 6 Department of General Surgery, The Fourth Affiliated Hospital, China Medical University, Shenyang, 110000 China; 7 Department of General Surgery Affiliated Zhong-da Hospital, Southeast University, Nanjing, 210009 China; 8 Beijing Institute of Respiratory Diseases, Beijing Chaoyang Hospital, Capital Medical University, Beijing, 100043 China

**Keywords:** Gastrointestinal diseases, Outcomes research

## Abstract

Venous thromboembolism (VTE) is an important postoperative complication. We investigated and analyzed the current inguinal hernia treatment methods and occurrence of early postoperative VTE in Chinese adults. This study involved data for patients with inguinal hernia hospitalized in 58 general hospitals in mainland China from January 1st, 2017 to December 31st, 2017. Data were retrospectively analyzed using a questionnaire. After data inputting and cleaning, we stratified and statistically analyzed patients' data using Caprini scores to create a high-, middle-, and low-risk group. A total of 14,322 patients with inguinal hernia were admitted to the 58 participating hospitals. After data collation and cleaning, 13,886 patients (97.0%) met our inclusion and exclusion criteria. The percentages of laparoscopic surgery and open surgery were 51.2% and 48.8%, respectively. 16 VTEs occurred during the hospitalization, accounting for 0.1% of all adverse events (95% confidence interval (CI) 0.11–0.13). The incidence of VTE was 0.2% (95% CI 0.18–0.2) in the high-risk group and 0.02% (95% CI 0.01–0.03) in the middle-risk group, based on Caprini scoring, with a significant difference (p < 0.0001). No VTE occurred in the low-risk group. Only 3,250 (23.4%) patients underwent Caprini risk assessment regarding treatment, with 13.2% receiving any prevention and only 1.2% receiving appropriate prevention. The treatment of inguinal hernia in Chinese adults has progressed somewhat; however, the evaluation and prevention of perioperative VTE was seriously neglected, in our study, and the incidence of postoperative VTE was underestimated postoperatively. Risk factors continue to be inadequately considered.

## Introduction

With developments in surgical techniques and materials, satisfactory results have been achieved in the diagnosis and treatment of inguinal hernia, worldwide^1^. Regarding the various aspects of the diagnosis and treatment of adult inguinal hernia, the HerniaSurge Group released the latest version of adult inguinal hernia management guidelines in 2018^2^. Along with the guidelines from several other international hernia associations, and other associations, the HerniaSurge Group guidelines discuss the problems related to the diagnosis and treatment of inguinal hernia, in different perspectives, among which, cited references included studies performed by Chinese researchers^3,4^. However, it is important to focus not only on improvements in surgical techniques and materials, but also on the importance of standardized perioperative patient management^5^. Among postoperative complications, venous thromboembolism (VTE) and pulmonary embolism are both potential and dangerous severe complications after inguinal hernia surgery^6^.

VTE affects approximately 5–15% of inpatients experiencing adverse medical or surgical events^7^. A large proportion of patients with inguinal hernia in China are older, and some have comorbidities. Additionally, the number of patients with inguinal hernia in China is extremely high, with an estimate of more than 1 million inguinal hernia operations being performed each year. Therefore, the total number of serious complications is considerable, including the risk of VTE^8^. In a prospective study published in 2015, in the immediate postoperative period, inguinal hernioplasty with mesh induces a temporarily slow venous flow in the ipsilateral CFV. However, this does not lead to an increase in the incidence of VTE^9^. In addition, to avoid postoperative hematoma or seroma, some doctors use postoperative inguinal compression, which may also lead to VTE clinically. Furthermore, a higher number of surgeons now choose laparoscopic techniques to treat inguinal hernia, both laparoscopy and general anesthesia have been considered to increase risk of VTE^10^. Even it is well known that a surgery itself creates the conditions for venous thrombosis. It seems that pleural undergoing laparoscopic inguinal hernia repair face a higher risk of venous thrombosis than open procedure.

The current medical environment and doctor-patient relationships in China are not ideal^11^, and although postoperative VTE is not associated with malignancy, it may be associated with considerable economic burden and patient danger, and surgeons' low awareness of the risk of VTE is not helpful. Despite the publication of the Surgical Branch of the Chinese Medical Association's "Guidelines for Prevention and Management of Perioperative Thrombosis in General Surgery in China" in 2016, Chinese hernia surgeons and general surgeons still pay insufficient attention to the occurrence of perioperative VTE, and it is very common for surgeons to have only a partial understanding of the perioperative management of VTE using anticoagulants^12^, especially regarding inguinal hernia. This is a very serious and common problem, and change is essential. The first step forward is awareness of the current situation. China still lacks a systematic and comprehensive patient follow-up database^13^; therefore, based on the platform of the Youth Committee of the Hernia and Abdominal Wall Surgery Committee of the Surgical Branch of the Chinese Medical Association, we designed a questionnaire to determine the prevalence of in-hospital early postoperative VTE and related factors in inguinal hernia surgery, and performed a retrospective cross-sectional study involving several general hospitals in China. We aimed to understand the current situation and related factors for in-hospital early postoperative VTE prevention and treatment of Chinese patients with inguinal hernia, and to provide practical basic data to support implementing the relevant guidelines.

## Methods

### Study sites

This was a multicenter, retrospective, cross-sectional study (ChiCTR1900020853). The study sites were 58 major hospitals in six different regions of China (east China, north China, northwest China, southwest China, northeast China, and central and south China, excluding Hong Kong, Macao, and Taiwan). All participating hospitals had more than 500 beds, which ensured optimal medical conditions and medical standards. All participating hospitals were able to perform inguinal hernia surgery.

### Patients and methods

The inclusion criteria were: (1) adult inguinal hernia patients, including those undergoing emergency operations, note that we also excluded outpatient, ambulatory operations. In this time era, very few outpatient hernia repairs were done at these 58 hospitals; and (2) admission between January 1st, 2017 and December 31st, 2017. We excluded patients who were younger than 18 years of age or who received early VTE-related treatment 24 h before admission.

The sample size was calculated based on an estimate of the number of inpatients with hospital-acquired VTE. Assuming that the lowest incidence of early VTE in inpatients was approximately 0.03, and the true prevalence of early VTE risk in 50% of inpatients was assessed with an accuracy of 5%, then at least 402 patients must be assessed using the Clopper–Pearson method, which required a sample size of 402/0.03 = 13,400.

We used a questionnaire to collect relevant data for all patients meeting the inclusion criteria. The questionnaire included demographic information, disease diagnosis, history of past illness, perioperative status, surgical details, risk factors, and the incidence of early VTE during hospitalization, and the questionnaires were completed by patients' surgeons. After summarizing the questionnaire data, we used EpiData 3.1 software (The EpiData Association, Odense, Denmark) to input the data twice. Logical proofreading was performed for the data inputting, and missing data and problematic data were sent to data acquisition personnel for proofreading.

### Data analysis

First, we used a Caprini risk assessment model^12^ to retrospectively assess patients' early VTE risk according to their preoperative conditions, and then divided the patients’ early VTE risk into three groups: low-risk, middle-risk, and high-risk. Patients' operative and perioperative management details were obtained from the questionnaire, and receiving VTE prophylaxis was classified as: no prophylaxis, any prophylaxis, or appropriate prophylaxis. Appropriate prophylaxis was defined as evaluating prophylaxis in accordance with the Guidelines for the Prevention and Management of Perioperative Thrombosis in General Surgery in China(intermittent compression devices, low dose heparin or heparin)^14^.

During data analysis, we performed descriptive analyses of the basic characteristics of all patients, as well as their early VTE risk; anesthesia and operation details; postoperative VTE prevention; and early VTE time distribution. All continuous variables were expressed as measured values, mean values, standard deviations, maximum values, and minimum values. Categorical variables were expressed as population numbers and percentages. The incidence of early postoperative VTE was calculated using a 95% confidence interval (CI). We used SPSS software version 23.0 (IBM Corp., Armonk, NY, USA) for all statistical analyses.

### Medical ethics

Our study was performed in strict accordance with the Helsinki declaration (1964) and the subsequent revised versions, and in accordance with the requirements of the Chinese Good Practice Guidelines, which are consistent with the Helsinki guidelines. The ethics committees of all sites approved the study protocol; the ethics committees review boards that approved our study also waived the need for informed consent because it was a retrospective study and the patient's data were anonymized. This study was approved by the ethics committee of Beijing Chaoyang Hospital, Capital Medical University and agreed by other 57 hospitals (ethics approval no. 2018-kd-315).

## Results

**Figure 1:**
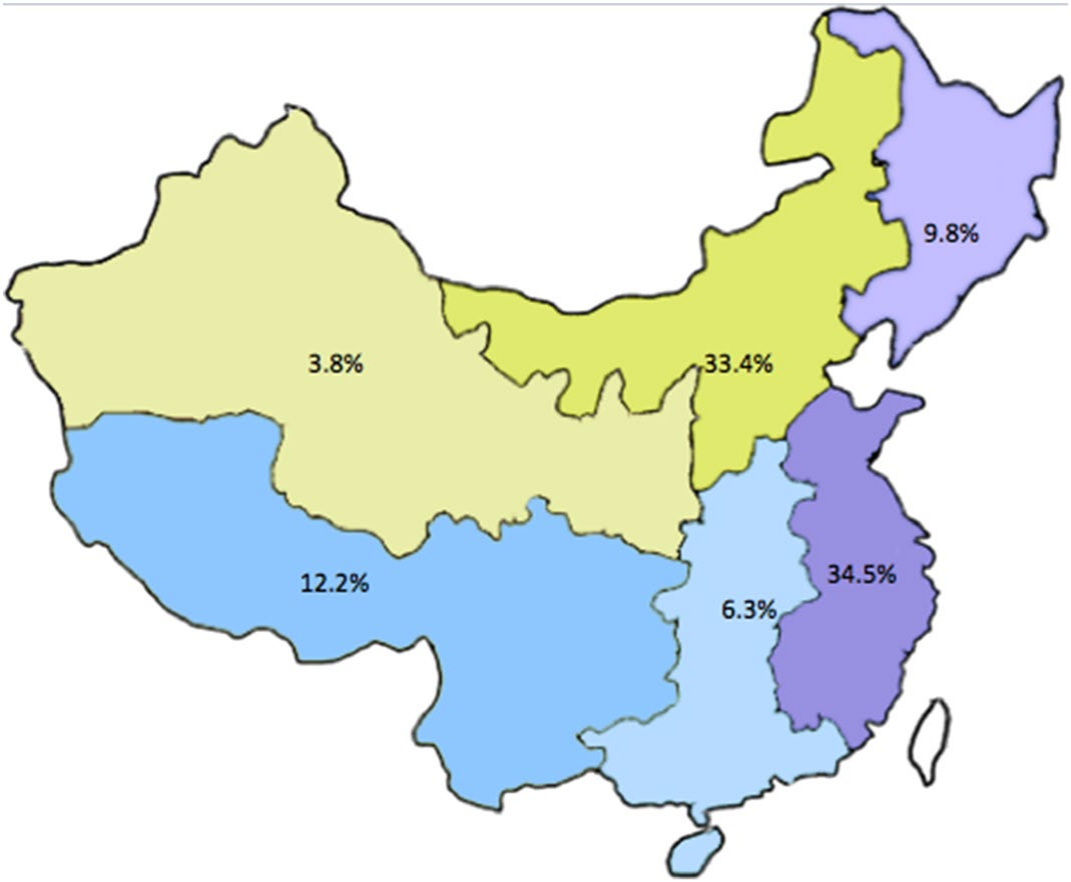
The source of clinical information from different areas in China.

Our research received a total of 14,322 questionnaires from 58 participating hospitals. After data cleaning and sorting, 13,886 (97.0%) complete questionnaires met the inclusion criteria. All cases were included into this study which met inclusion criteria in each center. We excluded 436 questionnaires that included data from patients aged < 18 years, were incomplete, or contained severe illogicalities and repeated data. Questionnaires included 4,643 (33.4%) from northern China, 4,788 (34.5%) from eastern China, 1,367 (9.8%) from northeastern China, 523 (3.8%) from northwestern China, 1,690 (12.2%) from southwestern China, and 875 (6.3%) from central and southern China (Fig. 1).

### Patients' background characteristics

Patients included 12,402 men (89.3%) and 1,484 women (10.7%) with an average age of 61.3 ± 16.7 years and a mean body mass index of 23.4 ± 3.1 kg/m^2^ (Table 1).

**Table 1:** Demographic data of all patients.

Project (n = 13,886)	Number (n)	Percentage (%)
**Age**		
18–40	1,989	14.3
41–59	3,452	24.9
60–74	5,311	38.2
≥ 75	3,134	22.6
**BMI**		
≤ 18.4	686	4.9
18.5–23.9	7,484	53.9
24.0–27.9	4,899	35.3
≥ 28	81	5.9
**Sex**		
Male	12,402	89.3
Female	1,484	10.7

### Medical history

There were 3,481 patients with hypertension (25.1%), 1,029 patients with diabetes mellitus (7.4%), and 1,016 patients with coronary heart disease (7.3%) (Table 2).

**Table 2:** Information in different groups classified by Caprini Scale^12^.

Project	Low-risk group (%)	Middle-risk group (%)	High-risk group (%)
**Complicated disease**			
Hypertension	87 (7.8)	1,093 (21.7)	2,301 (29.8)
Diabetes	34 (3.1)	357 (7.1)	638 (8.3)
Coronary disease	28 (2.5)	321 (6.4)	667 (8.6)
**Anesthesia**			
General	786 (70.6)	3,473 (68.8)	477 (61.9)
Intravertebral	44 (4.0)	357 (7.1)	659 (8.5)
Epidural	44 (4.0)	233 (4.6)	475 (6.2)
Local	239 (21.5)	983 (19.5)	1,814 (23.5)
**Operation method**			
Lichtenstein	121 (10.9)	1,534 (30.4)	1,896 (24.5)
Preperitoneal	1 (0.1)	779 (15.4)	1,618 (20.9)
Plug	0	83 (1.6)	528 (6.8)
Others of open	0	27 (0.5)	185 (2.4)
TAPP	673 (60.5)	1,837 (36.4)	2,783 (36.0)
TEP	317 (28.5)	761 (15.1)	702 (9.1)
Others of lap	1 (0.1)	25 (0.5)	15 (0.2)
**Laparoscopy (%)**			
51.23	89.04	51.98	45.3
**Prophylaxis**			
Instrument and drug (n = 129)	5 (0.5)	45 (0.9)	79 (1.0)
Instrument (n = 1629)	60 (5.4)	565 (11.2)	1,004 (13.0)
Drug (n = 73)	0 (0)	26 (0.6)	47 (0.6)
No prophylaxis (n = 12,055)	1,048 (94.2)	4,410 (87.4)	6,597 (85.4)

Instrument: static compression stockings.

Drug: low dose heparin.

### Risk assessment and thrombosis prophylaxis

According to patients' Caprini scores, 1,113 patients comprised (8.0%) the low-risk group, 5,046 patients (36.3%) comprised the middle-risk group, and 7,727 patients (55.7%) comprised the high-risk group. A total of 1,831 (13.2%) patients received any preventive measures and 12,055 (86.8%) received no preventive measures (Table 2); only 165 patients (1.2%) were treated with appropriate preventive measures.

### Surgical data

Among all patients, open surgery and laparoscopic surgery accounted for 48.8% and 51.2% of patients, respectively. Table 2 shows the distribution of Lichtenstein, preperitoneal open, mesh plug, transabdominal preperitoneal, totally extraperitoneal, and other surgical methods for inguinal hernia repair. The types of inguinal hernia confirmed intraoperatively were indirect inguinal hernia, direct inguinal hernia, and femoral hernia (Table 3).

**Table 3:** Classification of inguinal hernia.

Project	Number (n)	Percentage (%)
**Medical history**		
Primary	13,162	94.8
Recurrence	724	5.2
**Location**		
Left	4,551	32.8
Right	7,026	50.6
Bilateral	2,309	16.6
**Classification**		
Indirect hernia	10,622	72.5
Direct hernia	3,298	22.5
Femoral hernia	736	5.0

### Distribution of perioperative early VTEs

**Figure 2:**
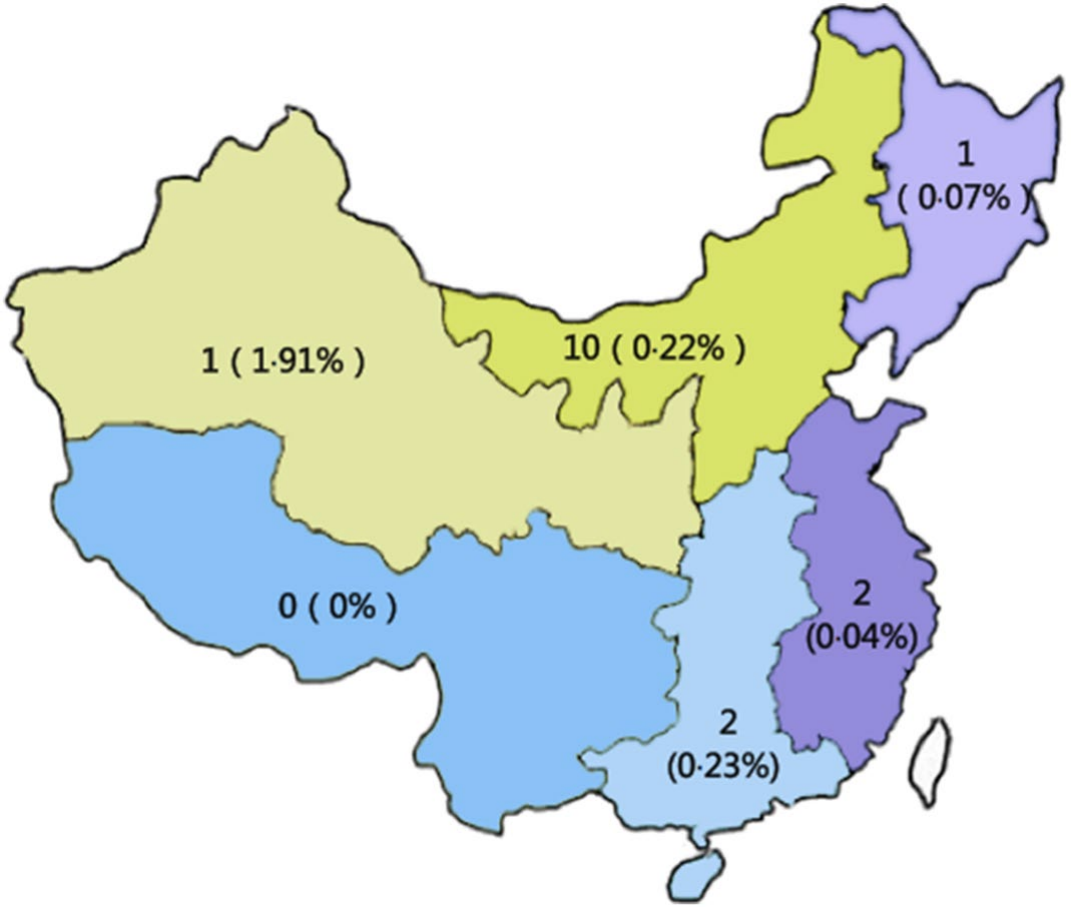
The incidence of early postoperative VTE in different areas.

Sixteen patients (0.12%, 95% CI 0.11–0.13) experienced early postoperative VTE after inguinal hernia surgery (Fig. 2). All 16 early postoperative VTE cases did not receive appropriate prophylaxis. Among these 16 patients, 4 had a previous history of VTE, 4 had a family history of VTE, and 2 had thrombophilias; these patients' average age was 69.2 ± 8.3 years. The operative methods were Lichtenstein in three patients (18.8%), preperitoneal open surgery in one patient (18.8%), mesh plug in one patient (6.3%), transabdominal preperitoneal technique in eight patients (50.0%), and totally extraperitoneal in one patient (6.3%). The ratio of open operation to laparoscopic operation was 43.8% vs. 56.3%. General anesthesia was used in 81.3% (13 patients) with early postoperative VTE, and local anesthesia was used in 18.8% (3 patients). The incidence of early postoperative VTE in the high-risk group with Caprini scoring was 0.2% (95% CI 0.18–0.20), significantly higher than that in the medium-risk group (0.02% (95% CI 0.01–0.03) (p < 0.001) (Table 4). No early postoperative VTEs were observed during hospitalization in 129 patients who received appropriate VTE prophylaxis.

**Table 4:** Distribution of early postoperative VTE events.

Project (n = 16)	Low-risk group (%)	Middle-risk group (%)	High-risk group (%)	p
Incidence	0	1 (0.02)	15 (0.2)	0.000
**Medical history**				
History of VTE (4)	–	–	4 (0.05)	
VTE family history (4)	–	–	4 (0.05)	
Thrombosis (2)	–	–	2 (0.03)	
No (6)	0	1 (0.02)	5 (0.06)	
**Anaesthesia**				0.001
General (13)	–	1 (0.02)	12 (0.16)	
Local (3)	–	–	3 (0.04)	
**Operation method**				0.001
TAPP (8)	–	–	8 (0.1)	
TEP (1)	–	–	1 (0.01)	
Preperitoneal (3)	–	–	3 (0.04)	
Plug (1)	–	–	1 (0.01)	
Lichtenstein (3)	–	1 (0.02)	2 (0.03)	

## Discussion

### Risk assessment and prevention of early postoperative VTE in patients undergoing inguinal hernia surgery in China has been seriously ignored

The focus of this study was to investigate the assessment and prevention of VTE in the diagnosis and treatment of inguinal hernia. After all the data were cleaned, we evaluated patients' data based on their Caprini scores and classified 36.3% of the patients as the middle-risk group and 55.7% of the patients as the high-risk group; these patients were at a high risk of experiencing thrombosis. However, only 3,250 patients (23.4%) underwent perioperative Caprini risk assessment, and the remaining patients had no data or incorrect data. Without correct patient evaluation, there is no proper prevention. Data showed that only 13.2% of patients in this study received any prophylactic measures, and of even more concern, only 1.2% received appropriate prophylactic measures. In our study, only a small number of middle- and high-risk VTE patients received correct prevention, indicating that Chinese general surgery/hernia surgeons appear to have the same problem with lack of awareness regardless of regional medical conditions and economic level. Even if surgeons used preventive measures, they often failed to comply with the recommended guidelines. Even more worrisome is that since 2017, China has gradually implemented an ambulatory surgical model; therefore, patients' hospital length of stay has decreased. Ambulatory procedures may actually decrease early VTEs because the patients ambulate more quickly. So, ambulatory inguinal hernia surgery was approved by the Chinese government in 2018 and was followed by a great deal of promotion. Patients now remain in the hospital for only 6–8 h during the entire treatment process. VTE is likely without proper assessment and prevention, and if doctors are unaware of the need for assessment and prevention, the situation is even more serious for high-risk patients leaving the hospital without this assessment.

### The incidence of early postoperative VTE after inguinal hernia is low and may be seriously underestimated

There are no large-scale studies in China to confirm the number of deaths caused by VTE after surgery. According to available statistics, VTE causes more than 296,000 deaths each year in the United States^15^, and the annual number of deaths secondary to VTE in the United Kingdom is more than 5 times that of breast cancer, AIDS, and traffic accidents^16^. Surgery itself is an important factor in the development of VTE. In a recent cross-sectional study of VTE assessment and prevention in inpatients in mainland China, data showed that only orthopedic surgeons in China had an understanding of VTE prevention. The emphasis placed by general surgeons on VTE was not up to the desired standard, and the proper preventive measures were implemented in only 2.1% of patients undergoing pelvic and abdominal surgery^12^. The 0.12% incidence of early postoperative VTE we saw in our study may actually be an underestimation for two possible two reasons: (1) our study was a retrospective study, and all patients did not undergo evaluation for thrombosis. Based on a large number of studies, it is reasonable to believe that a considerable number of asymptomatic early VTE patients are missed^17^; and (2) the highly-variable incidence period for VTE includes not only the perioperative period, but may extend to 1–3 months postoperatively^18^. With improved surgical techniques and medical technology in China, patients' hospitalization times following inguinal hernia surgery is now very short. Most patients leave the hospital within 48–72 h after surgery^19^. This study was a retrospective analysis based on inpatient medical records and included a relatively short observation time window, which may have led to missing some early postoperative VTEs. If VTE or pulmonary embolism occurs following hospital discharge, the consequences are often very serious.

A postoperative VTE-related study of inguinal hernia based on a European database was published in 2018 and included 28,782 patients undergoing elective inguinal hernia surgery. The study analyzed 90-day postoperative data for 2001–2011. The overall incidence of VTE was 0.18%, and the authors suggested that age, body mass index > 30 kg/m^2^, and hospitalization itself increased the risk of VTE^20^. The incidence of early postoperative VTE after inguinal hernia surgery has not been reported in Asia. Further studies are needed to confirm the exact incidence of early postoperative VTE after inguinal hernia repair in China.

### Older patients, herniorrhaphy, laparoscopic techniques, general anesthesia, history of VTE, and family history

In this study, patients aged ≥ 60 years accounted for approximately 60.8% of all patients, which was consistent with the incidence age of inguinal hernia^21^. Our data supported several high-risk factors for early postoperative VTE, including the inguinal hernia surgery itself. Performing surgery in the inguinal region slows blood flow velocity. By measuring the minimum blood velocity in the common femoral vein on the surgical side, flow velocity in the ipsilateral femoral vein was lower than that of the contralateral femoral vein (20.9 cm/s vs. 24.0 cm/s; p < 0.001)^9^. The diameter of the ipsilateral common femoral vein was larger than that on the contralateral side (p < 0.001), although this was found only with left inguinal hernia operation^9^. But in other publications such an observation has not been confirmed yet. Laparoscopic surgery requires general anesthesia which will increase the risk of VTE in elderly patients and possibly a controlled local anesthesia would be safer in elderly patients with serious comorbidities and high risk of VTE.

In our study, laparoscopic surgery accounted for more than half of the surgeries for inguinal hernia, which is likely related to the joint promotion of laparoscopic technology by our Chinese surgeons' academic organizations. Laparoscopic hernia repair technology for inguinal hernia treatment has improved, but these rapid developments bring both benefits and risks. Studies report that pneumoperitoneum and general anesthesia in laparoscopic surgery are risk factors for early VTE^22^, and our results showed that patients undergoing laparoscopic surgery and general anesthesia had higher rates of early VTE. More than half of our patients with early postoperative VTE had undergone laparoscopic surgery under general anesthesia (nine patients, 56.3%); therefore, surgeons must routinely assess the risk of VTE when choosing laparoscopic hernia repair. In addition, our patients with VTE with a history of VTE and those with a family history of VTE accounted for 50% of all patients with VTE. This result indicates the need to ask about a patient's family history of VTE because this may also be a risk factor for early VTE after inguinal surgery^23^.

In conclusion, treatment of inguinal hernia in Chinese adults has progressed somewhat; however, the evaluation and prevention of early postoperative VTE was seriously neglected, in our study, and the incidence of early VTE was underestimated postoperatively.

With our cross-sectional study, we hoped to show the current status of early postoperative VTE in patients with inguinal hernia in China, and call on surgeons to be aware of the need for evaluation and prevention of perioperative VTEs in patients with inguinal hernia. We also recommend establishing a simplified evaluation system or guidelines for thrombosis prophylaxis in patients with inguinal hernia in China. A simplified assessment system for thrombosis assessment has been established in obstetrics and gynecology in China. A similar system could be widely used in inguinal surgery to improve clinical efficiency and ensure medical safety by simple and convenient methods of evaluation and prevention.

Our findings may be of multifaceted significance: recommending and implementing the correct evaluation and prevention of VTE, attracting the attention of doctors and hospital administrators, and strengthening training and education in the prevention and treatment of VTE among surgical patients, and potentially all inpatients. We recommend a mandatory early postoperative VTE risk assessment and prevention system for hernia surgery, general surgery, and in hospitals, generally. We also recommend that the risk of thrombosis be minimized in patients undergoing all surgeries, not only those undergoing inguinal hernia repair, and in all inpatients, generally.

## Limitations

Our study has several main limitations. Firstly, All of our calculations of VTE were limited to the immediate time early postoperatively and actually inhospital which was a big limitation. Secondly, errors were present in the questionnaire entries, which required cleaning the data. Thirdly, questionnaire was filled out by surgeon instead of instead of a person blinded to the outcomes or the surgeon. Our participating hospitals were large regional general hospitals in China that were able to provide better medical services. The population is stratified into subgroups and we did not draw strong conclusions from such stratification. However, inguinal hernia surgery, as a common and frequently-occurring disease, is widely performed in primary-level hospitals all over China, and we did not investigate our objectives in the primary-level hospitals, which may have led to inaccurate data. Fourthly, the incidence of postoperative VTE was only calculated inhospital and not for the entire first 30 or 90 postoperative days after discharge. This may be one of the biggest limitations and it is best to describe it as the second limitation in the paragraph so that you do not look like you are trying to hide this limitation. Lastly, considering the population distribution and economic situation in China, some patients from underdeveloped areas and low socioeconomic areas are referred to developed areas, resulting in unbalanced data. Lastly, No discussion is present to describe how a diagnosis of VTE was made- symptomatic.

## References

[1] Access GH (2017). Healthcare Access and Quality Index based on mortality from causes amenable to personal health care in 195 countries and territories, 1990–2015: a novel analysis from the Global Burden of Disease Study 2015. Lancet.

[2] HerniaSurge Group (2018). International guidelines for groin hernia management. Hernia.

[3] Li J, Zhang W (2018). Closure of a direct inguinal hernia defect in laparoscopic repair with barbed suture: a simple method to prevent seroma formation?. Surg. Endosc..

[4] Access GH (2018). Measuring performance on the Healthcare Access and Quality Index for 195 countries and territories and selected subnational locations: a systematic analysis from the Global Burden of Disease Study 2016. Lancet.

[5] Moghadamyeghaneh Z, Hanna MH, Carmichael JC (2014). A nationwide analysis of postoperative deep vein thrombosis and pulmonary embolism in colon and rectal surgery. J. Gastrointest. Surg..

[6] Yang C, Zhu L (2017). Sudden death caused by acute pulmonary embolism after laparoscopic total extraperitoneal inguinal hernia repair: a case report and literature review. Hernia.

[7] Feng B, He ZR, Li JW (2013). Feasibility of incremental laparoscopic inguinal hernia repair development in China: an 11-year experience. J. Am. Coll. Surg..

[8] Gong W, Li J (2018). Operation versus watchful waiting in asymptomatic or minimally symptomatic inguinal hernias: the meta-analysis results of randomized controlled trials. Int. J. Surg..

[9] Lozano FS, Sánchez-Fernández J, González-Porras JR (2015). Slow femoral venous flow and venous thromboembolism following inguinal hernioplasty in patients without or with low molecular weight heparin prophylaxis. Hernia.

[10] Duranteau J, Taccone FS, Verhamme P (2018). European guidelines on perioperative venous thromboembolism prophylaxis. Eur. J. Anaesthesiol..

[11] Jing S, Liu S, Liu Q (2018). Impact of adverse media reporting on public perceptions of the doctor–patient relationship in China: an analysis with propensity score matching method. BMJ Open.

[12] Zhai Z, Kan Q, Li W (2019). VTE risk profiles and prophylaxis in Medical AND surgical inpatients. The Identification of Chinese Hospitalized patients’ risk profile for venous thromboembolism (DissvlVE-2)—a cross-sectional study. Chest.

[13] Poulose BK, Roll S, Murphy JW (2016). Design and implementation of the Americas Hernia Society Quality Collaborative (AHSQC): improving value in hernia care. Hernia.

[14] Geerts WH, Berqqvist D, Pineo GF (2008). Prevention of venous thromboembolism: American College of Chest Physicians evidence-based clinical practice guidelines (8th edition). Chest.

[15] Kahn SR (2013). Incidence of and mortality from venous thromboembolism in a real-world population: the Q-VTE Study Cohort. Am. J. Med..

[16] Humes DJ, Abdul-Sultan A, Walker AJ (2018). Duration and magnitude of postoperative risk of venous thromboembolism after planned inguinal hernia repair in men: a population-based cohort study. Hernia.

[17] Enoch S, Woon E, Blair SD (2010). Thromboprophylaxis can be omitted in selected patients undergoing varicose vein surgery and hernia repair. Br. J. Surg..

[18] Venclauskas L, Llau JV, Jenny JY (2018). European guidelines on perioperative venous thromboembolism prophylaxis: day surgery and fast-track surgery. Eur. J. Anaesthesiol..

[19] Solodkyy A, Feretis M, Fedotovs A (2018). Elective “true day case” laparoscopic inguinal hernia repair in a district general hospital: lessons learned from 1000 consecutive cases. Minim. Invasive Surg..

[20] Humes DJ, Walker AJ, Hunt BJ (2016). Risk of symptomatic venous thromboembolism following emergency appendicectomy in adults. Br. J. Surg..

[21] Simons MP, Aufenacker T, Baynielsen M (2014). European Hernia Society guidelines on the treatment of inguinal hernia in adult patients. Hernia.

[22] Ongen G, Yılmaz A, Cirak AK (2011). Venous thromboembolism risk and thromboprophylaxis among hospitalized patients: data from the Turkish arm of the ENDORSE study. Clin. Appl. Thromb. Hemost..

[23] Margaglione M (2013). Family history of VTE: an easy tool to score the individual risk. Thromb. Haemost..

